# Could saline instillation displace bacteria from the endotracheal tube biofilm to lower airways during suctioning procedure?

**DOI:** 10.1186/cc14689

**Published:** 2015-09-28

**Authors:** Anália Cristina CR Franchi, Antônio Pazin Filho, Camila Bottura, Carlos Henrique Miranda, Fernanda B Ferreira, Marcos de C Borges, Rodrigo de C Santana, Valéria T Okino

**Affiliations:** 1 Division of Emergency Medicine, Ribeirão Preto School of Medicine, São Paulo University, Centro, Ribeirão Preto, SP, Brazil

## Introduction

A biofilm is found on the inner side of endotracheal tubes (ETT) in mechanically ventilated patients. Saline instillation inside the ETT during the suctioning procedure is very common. This procedure could displace bacteria to the lower airways, increasing the risk of ventilator-associated pneumonia (VAP).

## Objective

Evaluation of the bacteriological cultures of the ETT lavage with saline after extubation of mechanically ventilated patients to verify dislocation of bacteria through this procedure.

## Methods

The ETT was removed using an aseptic technique during extubation. Saline (10 ml) was instilled into the tube, and the drainage fluid was collected on the other side. This material was sent to microbiological cultures in two different culture mediums (chocolate blood agar and MacConkey). We considered the quantitative culture of more than 100,000 UFC/ml as positive. The characteristics of the patients with and without positive cultures were compared.

## Results

Forty endotracheal tubes were analyzed (*n *= 40). Positive culture was observed in eight tubes (20 %). The bacteria observed were: five Gram-positive (*Staphylococcus aureus *in three, *Streptococcus pneumoniae *in one and *Staphylococcus haemolytics *in one) and three Gram-negative (*Acinetobacter baumani, Klebsiella pneumoniae, Enterobacter cloacae*). We did not observe differences between the group with positive and negative cultures in relation to demographic and clinical characteristics, intubation time and tube diameter (*p *>0.05).

## Conclusion

The use of saline during an endotracheal suctioning procedure can dislocate pathogenic bacteria from the endotracheal tube biofilm to the lower airways, and could increase the risk of VAP. The use of saline should be minimized during patient care.

